# Dr. Gaston Explains

**Published:** 1887-07

**Authors:** J. McF. Gaston


### DR. GASTON EXPLAINS.

*Editors Atlanta Medical and Surgical Journal:*

The suggestion in my communicaiion of last month, that there ought to be two additional commissioners for the proper investigation of yellow fever inoculation, was sustained by a large majority of a full meeting of the members of the American Medical Association at Chicago. A motion to lay the resolutions on the table was voted down, and after their passage a motion to reconsider was rejected. Subsequently, when comparatively few were in attendance, a motion by a government official was carried through by parliamentary tactics to reverse this decision of the Association. It is highly probable that nothing would have been accomplished with the opposition of the President's medical adviser. But the sense of the profession has been clearly expressed, and is not changed by this vote.

A paper was in the hands of the Secretary when this action was taken, announcing the names of the committee under my resolution, as follows:

J. McF. Gaston, M. D. Atlanta, Ga.; William Brodie, M. D., Detroit, Mich.; J. J. Chisolm, M. D., Baltimore, Md.

The memorial, in compliance with the resolutions, was drawn up to be signed and forwarded by this committee, recommending Dr. J. L. Guiteras, of Charleston, S. C., and Dr. H. M. Lane, of Carthage, Mo., as additional commissioners.

These two competent medical men have been suggested by me, and I trust this, with the avowal in my former letter, may confirm the impression of my interest in this matter. All who are aware of the agency I have had from the outset in urging forward this investigation should understand that it has been advocated as a measure of great public utility and of prime necessity for the arrest of yellow fever. The opportunity to get at the facts connected with the experiments in Brazil, through correspondence with Dr. Freire, and the documentary evidence furnished to me from other sources as to the developments in Mexico, Columbia and Panama, have kept me informed on this subject.

If the investigations of Dr. Sternberg are extended so as to elicit the testimony which is availible in support of the inoculability of small-pox and the protection afforded by it, all will be solved satisfactorily in regard to yellow fever inoculation. But the voice of the profession has gone forth in tones which cannot be mistaken in favor of the addition of two other members to the commission, and should the investigation miscarry for lack of this precaution, then let the responsibility rest where it belongs, and not upon those who have recommended this measure.

J. McF. GASTON, M. D.

